# "The Hospital" Nursing Section

**Published:** 1906-04-14

**Authors:** 


### The Hospital.

**Nursing Section.**

Contributions for "THE HOSPITAL," should be addressed to the EDITOR, "THE HOSPITAL" NURSING SECTION, 28 & 29 Southampton Street, Strand, London, W.C.

No. 1,020.—VOL. XL. SATURDAY, APRIL 14, 1906.

### Notes on News from the Nursing World.

### RETIREMENT OF THE MATRON OF ST. MARY'S HOSPITAL, PADDINGTON.

A GREAT loss is about to be sustained at St. Mary's Hospital, Paddington, by the retirement of the matron, Miss Medill, after a service of 21 years. The fact was first made public by the Chairman, Mr. H. A. Harben, at the general meeting of the Paddington Branch of the St. Mary's Hospital Ladies' Association last week, when both he and Lady Dimsdale, President of the Association, expressed high appreciation of Miss Medill's great services to the hospital, and their deep regret at her retirement—expressions which were very warmly endorsed by the large gathering of friends of the hospital present on the occasion. Miss Medill received her training at the Middlesex Hospital, became sister at St. Bartholomew's, and then lady superintendent of the East London Hospital for children. The last post she held for three years, being appointed in March 1885 to the important post which she is now relinquishing. Some idea of the changes in hospital nursing which have taken place in that period may be obtained from the mere comparison of the numerical strength of the nursing staff of St. Mary's when Miss Medill entered upon her duties and at the present time. Then a staff of 40—9 sisters and 31 nurses—was thought sufficient for 244 beds; now for 281 beds the staff numbers 108, equal to one nurse to less than $2\frac{3}{4}$ beds, and this is felt to be inadequate. Throughout this period of development Miss Medill has kept before her nurses the highest ideals, and has consistently sought to advance their interests and to secure their well-being in every way. In April 1900 the time of training was altered from three to four years, in the interest of both the hospital and the nurses. Miss Medill considers that the extra year is beneficial to the nurses because after the wear and tear of theoretical instruction is over, with increased knowledge they can go on so much better with the whole subject. Another change which was initiated at the same time was to alter the trial period of service in the wards from one to three months. This, as the matron anticipated, has proved satisfactory. Having seen her nurses housed in their new Home in the Clarence Wing, and having taken the opportunity which that instalment afforded to secure for them the removal of many disadvantages under which they formerly laboured, the result directly and indirectly of the poor accommodation hitherto available for them, Miss Medill, while still well and strong, alert and active, and sensible of no failing powers, steps down from her place. The Board of Management, the medical staff, her colleagues in the administration, and, above all, St. Mary's nurses, who know the whole-hearted devotion with which she has served the hospital she loves so well, will join in the hope that years of happiness lie before her.

### NURSES' QUARTERS AT THE WEST LONDON HOSPITAL.

THE accommodation for the nursing staff of the West London Hospital has been improved by the adaptation of two adjoining houses. This extension was rendered necessary on account of some of the nurses' rooms in the hospital having been done away with in order to make a new corridor. Of those that remain in the hospital most of the bedrooms are far from satisfactory, the windows being almost entirely blocked by the erection of a new ward, so that the ventilation leaves much to be desired. Urgent demands on space require three beds in rooms where there should only be two. Even in the new houses it would be far better if all the rooms could be single-bedded instead of double-bedded as they must be now. Here comes in the inconvenience of adapting private houses for a purpose for which they were never intended, though if a third house could be acquired so that each nurse could have a separate room, the arrangement would be more satisfactory. The rooms in the new houses are bright and airy and well fitted with cupboards, so essential to comfort and convenience! One house is set aside for the night nurses, and as all the domestic work has to be finished by 10 A.M., the night nurses need fear no disturbance of any kind from the outside world. The night sister has charge of this house and the assistant matron of the other. There are three bathrooms for the 26 inmates, a constant supply of hot water day and night, and in one of the kitchens a fire is kept burning all day on Saturday, in order that the airing of clothes and sundry washing arrangements can be indulged in by the nurses. By throwing the two small gardens belonging to these houses into one, a piece of ground has been obtained, which will be a boon to the nurses who have hitherto had no place to sit in save the hospital garden frequented by the patients.

### THE TRAINING AT FIJI COLONIAL HOSPITAL.

IN his annual report on the training school for nurses, conducted by the Government of Fiji at the Colonial Hospital, Suva, the chief medical officer states that the lectures delivered by the medical and surgical staff were 68 in number, including eight anatomical demonstrations, of which six were in general anatomy, on the cadaver, and two were in superficial anatomy on the living subject. Besides these, 24 lectures were delivered by the matron: two on ward routine, five on operation duties and after-care, fourteen on practical nursing, and a course of three sittings on ethics, which were in the nature of conversation rather than formal lectures. In addition the dispenser gave three months' class teaching and practice in pharmacy. The custom at the Fiji Hospital is to divide the year into three terms or sessions of three months each, leaving a month's interval between each term. The terms begin on the 15th of January, May, and September, and a time-table, based on the permanent scheme and syllabus, is made out for each term successively, in such a manner as to be applicable to the several pupils, each in her own stage of progress. The pupils, therefore, know beforehand what range of subjects they will be expected to study during each term, and can accordingly take their off-duty leave during one or other of the intervals between terms without sacrificing any lectures or demonstrations. No doubt it is in consequence of this admirable arrangement that during the whole of 1905 not a single pupil missed a lecture nor was absent from class teaching at the appointed times.

### IMPERIAL MILITARY NURSING SERVICE.

We are officially informed that Miss A. L. Cox, matron in Queen Alexandra's Imperial Military Nursing Service, has been transferred to the Military Hospital, Shorncliffe, from the trooping duty steamship *Plassy*. Miss E. J. M. Keene, sister, has been transferred to the Royal Infirmary, Dublin, from the Military Hospital, York; Miss L. M. Culverwell, sister, to the Military Hospital, York, from the Queen Alexandra Military Hospital, Millbank; Miss S. Smyth, sister, to Cambridge Hospital, Aldershot, and Miss S. K. Bills, sister, to the Queen Alexandra Military Hospital, Millbank, from the trooping duty steamship *Plassy*. Miss E. Barber, staff nurse, has been transferred to the Military Hospital, Gibraltar, from the Military Hospital, Malta; and Miss E. M. Fairchild, staff nurse, to the Royal Victoria Hospital, Netley, from the trooping duty steamship *Plassy*. Miss M. Steenson has arrived from South Africa.

### SCHOLARSHIPS FOR STUDENTS OF MIDWIFERY.

We are officially informed that the London County Council is prepared to award twelve scholarships a year to students in midwifery, six in July and six in January. Candidates must reside within the county of London, must furnish satisfactory evidence as to character, must be between 24 and 40 years of age on July 31, 1906, and must be certified by the Council's medical officer as being in good health. They are also required to satisfy the Council as to their need for pecuniary assistance for the purpose of training as midwives, to enter into a bond with sureties that they intend to practise as midwives for two years within the county of London, to pass a qualifying examination in writing from dictation and elementary arithmetic, the standard being substantially equivalent to that of Standard VI. of the elementary school code, which can be obtained for threepence from Messrs. Wyman and Sons, Fetter Lane, E.C. It has been decided that preference may be given to candidates who produce evidence of satisfactory training in general nursing or of practical experience of sick nursing among the poor. The value of the scholarship is to be £25, and this sum will be paid to the institution selected by the applicant in part discharge of the fee for board, lodging, and tuition. The course of training, including practical work at the institution, will extend over a period of six months, and scholarships must be held at one of the four institutions approved by the Council and recognised by the Central Midwives Board—namely, Queen Charlotte's Lying-in Hospital, the East-end Mothers' Lying-in Home, Clapham Maternity Hospital, and the Central Lying-in Hospital, York Road, Lambeth. Forms of application for the scholarship may be obtained from the Education Department of the Council, Victoria Embankment, W.C., and must be returned to the same address not later than Saturday, May 5. The qualifying examination will take place on Friday, May 25, and the first award will be made in July.

### THE WORKHOUSE MASTER AND THE THERMOMETERS.

It will be remembered that a few weeks ago the Exeter Guardians congratulated themselves on the fact that they had invested the master of the workhouse with power to regulate the temperature of the wards in the Workhouse Infirmary. Here is the sequel. At a meeting of the Guardians last week a report from the visitors that the temperature in some of the wards was too low, and the master was requested to explain. His explanation was that the medical officer and superintendent nurse were responsible for the temperature, that it was out of his province, and he added: "The instruction of the Board was that I should see that the thermometer did not exceed 65°. No minimum was laid down, but if there had been, I should have interfered." Could anything be more ridiculous than this? The Guardians now probably realise that our insistence upon the medical officer and superintendent nurse having the entire control of the infirmary wards was not so unreasonable as they imagined. Meanwhile the matter has its serious aspect. If any of the patients suffered from the temperature of the wards being too low, because the Guardians ordered the master of the workhouse to exercise authority, they would be held responsible for the results.

### A DIMINISHING STAFF AT SALISBURY.

The difficulty of maintaining a sufficient staff of nurses was discussed at the annual meeting of the Salisbury Nurses' Home. The Chairman pointed out that according to the report for 1905, the adoption of which he had been desired to move, the present number of nurses was only twenty-four, whereas it has been as high as forty-two. The supply, it seems, has been gradually diminishing, and the

Committee are naturally anxious that this should not go on. In fact, if the calls on the Home are to be met there must be an increase. Formerly, all the nurses attached to the Home were trained at Salisbury Infirmary, and there is now a suggestion that some plan of the kind should be reverted to. As the nurses receive from the Home nearly £50 per annum, including payments on their behalf to the Royal National Pension Fund, to which the Institution is affiliated, and the lady superintendent, Miss Laurence, is a very able manager, there ought not to be any trouble in keeping up the adequate strength of the staff.

### THE INFLUENCE OF CONTACT WITH SUFFERING.

The *Christian Commonwealth* has opened its columns to a correspondence on the question "whether professional nursing hardens." The balance of opinion appears to be in the negative, and the testimony of a correspondent whose husband was a patient in St. Bartholomew's Hospital for nearly two months altogether supports this view. There is no doubt whatever that frequent contact with suffering has one of two results. It either hardens or softens, and its effect upon different nurses indicates, more or less, how far they are qualified for their work. If constant tending of the sick does not materially increase sympathy with the sufferers, it proves that the nurse has mistaken her vocation. On the other hand, a true nurse, witnessing the agony borne by her patients, cannot fail to have drawn out all the finer qualities of her nature, and the more these qualities are developed the more her ministrations will be appreciated.

### OUR PRIVATE SALE AND EXCHANGE COLUMNS.

We direct atention to a new departure which has been made by the manager of The Hospital for the convenience of nurses and other advertisers. A certain number of columns are now specially reserved for the private sale and exchange of personal and miscellaneous articles. In order that the name and address of the advertiser need not be published, a number is attached to each advertisement and replies addressed to this number are forwarded free of charge. With the view of rendering the department as useful as possible, only a small charge is made for advertisements, and to facilitate negotiations the manager is willing to receive the price of any article from prospective purchasers of goods advertised, and to hold the money until the purchase has been completed or the article returned to the advertiser. This, it will be observed, constitutes a practical guarantee against loss. Nurses, we think, will find these columns advantageous to them when they desire to purchase, sell, or exchange, uniforms, appliances, books, or magazine literature.

### AN ADDENBROOKE'S NURSE DROWNED.

On Monday morning two of the nurses at Addenbrooke's Hospital, Cambridge, engaged a Canadian canoe for a trip up the river Granta. When they reached the stretch of the river which is called Paradise the canoe capsized, and both the occupants were upset. One of the nurses, Miss Shipworth, managed to cling to the upturned craft, and was ultimately dragged ashore by means of ropes; but the other, Miss Aldridge, had disappeared, and when her body was recovered life was extinct. We sympathise with the authorities of Addenbrooke's, as well as with the relatives and friends of the unfortunate nurse who was drowned, in respect to a calamity which, of course, they were powerless to avert.

### DISCLAIMERS FROM NURSES.

It will be seen from our correspondence columns that two nurses, each bearing the name of Alice Nicholls, are alarmed lest any one should connect them with the person who was recently convicted of theft at Bow Street Police Court. We gladly publish their letters, but we do not think that they have any cause for anxiety. Those who know them will not confuse them with the Alice Nicholls who is suffering the penalty of her offence, and for the rest it can scarcely matter. One of our correspondents mentions that she once stayed at the Nurses' Hostel, but as she has not been out of Brighton since January, she is not likely to be misjudged. The incident, however, illustrates the far-reaching consequences of misconduct, and we can quite sympathise with the feeling that prompted disclaimers from the innocent.

### CHANGES AT BARROW.

At the last meeting of the Barrow Guardians the resignation of two nurses was announced, and one of the Guardians said it was "deplorable to find their nurses continually leaving." The rejoinder was that both were going in order to improve their position, and the Chairman claimed that the fact was a credit to the Barrow Union. We agree with the Chairman on this point, but if, as it was stated, 15 or 16 nurses have left Barrow Workhouse during the last three years, we can hardly suppose that they all quitted in order to benefit themselves. Such frequent changes cannot, at any rate, be for the benefit of the institution.

### AN ADMIRABLE BEQUEST.

We learn with interest that under the will of the late Mrs. Edith Frances Stopford Sackville, the sum of £2,000 is to be invested for the purpose of providing a nurse for the sick poor of Fowey. This is a somewhat unusual kind of benefaction, and we should be glad to see it more frequent.

### £12 A YEAR FOR AN ASSISTANT NURSE.

The St. Asaph Guardians have been advised by the Local Government Board Inspector for the district to appoint "another trained nurse" at the workhouse, the nursing in that institution being performed at present by one nurse, they decided to advertise for an assistant nurse at a salary of £12 a year. At the last meeting a guardian protested that if they offered such a salary they would be "the laughing-stock of the whole country." But his protest was curtly set aside by the Chairman, who rejoined that the question of salary had been settled and could not be further discussed. We need hardly say that it is happily quite impossible to obtain the services of a nurse with any training worth speaking of for the salary fixed by the St. Asaph Guardians.

### The Nursing Outlook.

"From magnanimity, all fears above;
From nobler recompense, above applause,
Which owes to man's short outlook all its charm."

### NURSES' SOCIAL UNIONS.

THE county of Somerset has led the way in a matter which is worthy of the close attention of all who have the welfare of the working nurse at heart. Somersetshire has been mapped out into seven centres, each of which has its own organiser. The organiser makes it her business to get into touch with all the nurses working within her centre, and aims at gradually becoming personally acquainted with as many of them as possible. It is her aim to see that each nurse is kept acquainted with the new developments of her profession, that she has an opportunity of meeting other nurses and others interested in their work, and that those nurses who are solitary or isolated are provided with friends and recreation. This is all good, but the principle which underlies the Nurses' Social Union is the best part about it. That principle makes it the aim of all connected with the organisation to maintain a high ideal of work and thought.

At first sight it might be thought well nigh impossible to accomplish these objects effectually. In practice Miss E. L. C. Eden, the founder of the Union, has so carefully thought out her scheme that the initial difficulties have been speedily overcome. Each organiser arranges a meeting three or four times a year of the nurses working within her centre. There are seven centres. These meetings require the co-operation of some of the residents in the district, who lend their houses and undertake to provide tea and light refreshments. The aim invariably is to make each meeting of a simple, homely character, and as sociable as possible, with little or no expense whatever to the nurses themselves. Of course in counties there are many district nurses, who are best able to attend in the afternoons, and the hour of meeting is fixed to suit their convenience. In addition to the quarterly meetings it has been the practice every three years or less to have a large fête at some country house in the county, which all the nurses are invited to attend. In this way no nurse in the county is beyond the reach of friends and professional help. Many nurses too may be within the reach of meetings in more than one centre, as the aim is to choose places in such a way as to secure that every part of the centre shall hold a meeting in due course. Nurses are at liberty to attend gatherings in their own centres, or in a neighbouring one, should they wish to do so. It is the organiser's privilege with her assistants to introduce nurses to each other, and to arrange that the whole of the nurses in a centre in this way become acquainted. Sometimes the meetings are wholly social in character, but the organisers endeavour to find speakers at others, either doctors or educated nurses, who deliver an address or read a paper on some professional subject. In this way post-graduate instructions can be given with great advantage to district nurses especially. It is interesting to note that the Social Union embraces every kind of nurse—hospital, private, district, infirmary, and workhouse nurses are all brought into touch, but especial care is taken to help the lonely worker and those who are shut off from the stimulus of hospital or town life.

The movement has attracted the notice and has secured the support of the managers of the principal hospitals in the county. Miss Eden, for instance, was gratified by an invitation from the matron and committee of the General Hospital, Bristol, to bring all the members, including nurses from many rural districts, to the hospital, where they were received with great hospitality and passed a most pleasant and profitable afternoon. The visitors were shown all over the hospital, and after tea an excellent and practical demonstration of the *x*-rays and Finsen light was given by Dr. Kenneth Wills. We have no doubt that the excellent example set by the matron and committee of the General Hospital, Bristol, will be generally followed, and that in this way, in addition to the social advantages of the Union, its practical usefulness for post-graduate purposes will be developed considerably. How much these gatherings of the Social Union are appreciated by the nurses is indicated by the fact, that on a recent occasion one nurse walked ten miles to the meeting, and Miss Eden's correspondence shows how helpful they have proved to many nurses, and how much the nurses have enjoyed them.

The success which has attended Miss Eden's scheme and its practical character are proved by the fact that Miss Amy Hughes has accepted the office of President, and that Miss Eden has secured a most able assistant in Miss Joseph. Why should not each county have a Nurses' Social Union? Wherever one is started it should be provided that the heads of the nursing profession in the county, and the managers of the hospitals within it should be made honorary members. The committees consist of the local organisers *ex officio*, of representatives of the nurses—two from each centre—and of co-opted members, mainly resident in the county, who have shown a practical interest in the work. In Somersetshire it has been working for five years, and is now firmly and permanently established. One useful purpose of the Union is to organise a library of professional books and papers, so that every nurse may have an opportunity of keeping up with her work. A loan collection of nursing appliances is also maintained in connection with the Union. We congratulate Miss Eden upon the marked success which has attended her efforts, and the good and useful work which these Unions are capable of accomplishing in counties.

### Abdominal Surgery.

By Harold Burrows, M.B., F.R.C.S., Assistant Surgeon to the Seamen's Hospital, Greenwich, and to the Bolingbroke Hospital, Wandsworth Common.

### AFFECTIONS OF THE STOMACH.

### Gastric Ulcer.

Ulceration of its mucous membrane is the most important disorder to which the stomach is liable, and the nursing of gastric cases might be dealt with almost completely under the heading of gastric ulcer and its consequences.

Gastric ulcers may be classified as malignant and non-malignant. The latter form will be dealt with first.

### Simple Ulcer.

Non-malignant, or, as they are commonly called, "simple" ulcers of the stomach, are usually met with in adults between the ages of 20 and 40, although they are not confined to this age period. They occur especially in anæmic girls, and in men who customarily drink more alcoholic beverage than is good for them. The course which a gastric ulcer runs, and the complications to which it gives rise, vary within wide limits. Generally, these ulcers may be divided into acute and chronic. The distinction is of importance chiefly in connection with the complications, which differ somewhat in the chronic and the acute cases.

### Symptoms.

The leading symptom is pain in the stomach after taking food. The pain may come on immediately after food or not for an hour or perhaps two hours, but the characteristic feature is the constant repetition of the pain after every meal; and, as a rule, in each case there is some constancy in the lapse of time between the taking of food and the onset of pain. The pain may be very slight, perhaps described merely as a "burning sensation" or a "feeling of emptiness," or it may be so intense as to lead the patient deliberately to starve himself for fear of the torment which food produces.

Vomiting is the next noteworthy symptom; and it is commonly followed by speedy relief of the pain.

Usually there are in addition anæmia, tenderness to pressure in the epigastrium, loss of weight—if the ulcer has existed for a long time—and excess of hydrochloric acid in the gastric juice as shown by chemical analysis of the stomach contents after a test meal.

Rarely one of the serious complications of gastric ulcer, such as hæmorrhage or perforation, is the first recognisable evidence of the ulceration.

The symptoms have to be distinguished from those of indigestion caused by unsuitable and improperly prepared food or by insufficient mastication, and from over-secretion of hydrochloric acid without ulceration (hyperchlorhydria).

### Treatment.

The treatment of every case of gastric ulcer must be energetic and determined from the moment the diagnosis is made. The patient must be kept at rest in bed, an endeavour made to lessen the anæmia, the feeding most carefully arranged and carried out, and throughout a vigilant watch kept in order to detect at once the earliest signs of any complication.

Before being put to bed the patient should be weighed, and his weight recorded for future reference.

With regard to the anæmia, experiments on animals have seemed to show that this plays an important part in favouring the development and persistence of gastric ulcer; so that there is a special indication for treating the condition of the blood. This is not always quite simple to do in a satisfactory manner on account of the severe dietetic restrictions which may have to be imposed upon the patient. Yet it is possible to achieve some benefit. Fresh air, regulation of the bowels, the administration of soluble preparations of iron, and other drugs are available for the purpose. Of these fresh air is not the least potent. If the patient can be wheeled on to a balcony, this should be done; otherwise the nearest approach possible to getting his bed out of doors should be attained.

These methods of treatment, however, are subsidiary to the proper regulation of the feeding of the patient. In order that the stomach may heal it should be at rest, and obviously it cannot be at rest so long as it contains food which it has to digest and to force through the pylorus into the duodenum.

In some cases it may be necessary to prohibit for a time all food by the mouth, the patient being sustained on nutrient enemata, accompanied in occasional instances by subcutaneous feeding. In other cases a certain amount of food may be allowed by the mouth. In this event the food must be liquid in form, small in quantity, and given at regular intervals. If it causes no pain the amount may be cautiously increased. Pain and vomiting after food are indications for reducing the amount or for stopping it altogether. When the epigastric tenderness has quite disappeared, and the patient can take ordinary food without pain or discomfort the ulcer may be regarded as cured. Nevertheless care must be exercised subsequently as to diet, indigestible food being avoided and the meals taken at regular intervals. A recrudescence of the pain and other symptoms of ulcer may occur, and should be met by immediate treatment as before.

### Complications and their Treatment.

The chief complications of gastric ulcer are hæmorrhage, perforation, gastric adhesions, dilatation of the stomach due to obstruction, and subphrenic abscess.

*Hæmorrhage.*—It is easy to imagine how an ulcer may erode a blood-vessel in the stomach wall and cause hæmorrhage. With a chronic ulcer a little blood is often to be found in the gastric contents, but a copious hæmorrhage is exceptional, perhaps because the process of clotting in the blood-vessels keeps pace with the process of ulceration.

It is mostly with acute ulcers that large hæmorrhages occur, and in these cases it may be presumed that the ulceration has extended so quickly that a vessel has been opened before the blood within it has had time to form a firm clot.

When small hæmorrhages ensue, as in chronic ulcer, the blood appears in the gastric contents as a brownish material, the colour having been altered by the gastric juice. In copious hæmorrhage the blood may be bright red. Sometimes a patient loses a large quantity of blood without vomiting, and in these cases it is easy to overlook the hæmorrhage unless a careful look-out be kept for melæna (black stools), remembering that certain drugs (bismuth, iron) cause an appearance somewhat similar to true melæna.

In the case of a severe attack of hæmatemesis, the patient should be kept lying on his back and told to remain quite still. Of course, he will be frightened, and, above all, he should be calmed with the assurance that there is no danger and that the bleeding will certainly cease before long, for a restless patient and a tumultuously beating heart are not favourable to the arrest of hæmorrhage. A hot bottle should be placed at his feet, and an ice-bag applied to the epigastrium. Nothing should be given by the mouth except with the doctor's advice. The doctor may give an injection of morphia to quiet the patient, or an injection of ergotin, and he may like him to have lumps of ice to suck. Transfusion apparatus should be in readiness in case it may be required. In rare instances it may be thought necessary to perform an emergency operation, with the object of opening the stomach and securing the bleeding vessel. As a matter of fact, with proper treatment short of operation these hæmorrhages are seldom fatal.

### The Nurses' Clinic.

### THE DISTRICT NURSE AND ERYSIPELAS.

Erysipelas is happily much less common than it was even twenty years ago, but its occurrence, whether in obvious connection with wounds or otherwise, is always possible in unhealthy surroundings. The first point to be noted is its extremely infectious nature, and the especial danger of allowing any pregnant woman to come into direct or indirect contact with it, or any person suffering from a wound, however small or insignificant. Whatever inconvenience or loss of time it may cost her, the nurse must visit the erysipelas case last on her round both morning and evening; special sleeves and aprons must be left at the patient's house; her hands must be thoroughly cleansed, her throat gargled with carbolic, and the thermometer disinfected. At the termination of the case the nurse should have a bath with one tablespoonful of izal to 30 gallons of water, and her hair and clothing should be disinfected. A stronger solution of izal is sometimes recommended, but frequently causes a rash. If there is only one district nurse in the neighbourhood it may be difficult, or even dangerous, for her to undertake a case of erysipelas, but it must be remembered that the patient is in great need of help, and that he would be peculiarly unwelcome in any modern hospital. If, however, the district nurse undertakes midwifery, or even monthly nursing, she is absolutely unable to attend erysipelas. Cellulitis is of the same nature, but attacking the deeper tissues, is equally infectious, and demands the same precautions.

The second point is the acute and dangerous nature of the disease. In all surgical cases the nurse must take the temperature at each visit; a sudden rise of temperature, with a blush round the wound of a smooth and continuous nature, always suggest erysipelas. If there is no open wound, and erysipelas appears on the face, it usually begins at the nostrils, the corners of the mouth, or the eyelids. Very high fever and violent delirium are more likely to happen when erysipelas occurs on head and face than when it is found on limbs, scrotum, or anus. Erysipelas is most deadly in the case of patients who are very old, very young, or of intemperate habits.

The third point to be remembered is that medical opinion differs greatly as to details of treatment of this disease, and therefore no district nurse should wash the affected part, cover the face with a mask, or use any ointment or powder until she has received definite instructions from the doctor in charge of the case. It may be said that the general treatment is to exclude air from the surface affected by the aid of powder or ointment and cotton wool, but very different applications are used to achieve this purpose.

Erysipelas most commonly affects the head and face, which become almost unrecognisably swollen and disfigured. A large lint mask tied behind and held in place by a triangular bandage is generally used, the face being first covered with a mixture of equal parts of ichthyol and lanoline painted on and covered with gauze; or a mixture of iodoform and lanoline (four parts to ten) protected by lint and oiled silk; or zinc ointment. Treatment always begins with a purgative, usually calomel. Erysipelas is especially likely to attack those who have lived in hot climates and developed weakness of liver or kidneys.

There is a heavy discharge from eyes, ears, and nostrils. The eyes must be bathed with warm boracic lotion at least every two hours, and the doctor will probably order ears and nose to be syringed, and the patient must gargle with chloride of potash. It is during this part of the treatment that the attendant is in most danger of contracting the disease.

If the patient wears a mask, there is some difficulty with regard to feeding, and the nurse should ascertain that the relatives understand how to use a feeder, or a feeder with a tube attached. Nourishing and sufficient food is of the first importance.

The temperature will be very high, and there may be delirium, possibly of a violent nature, and proper precautions must be taken.

Death frequently occurs about the fourteenth day, and if the patient tides over that period purpura often supervenes. When this is surmounted a large abscess may form in the hip-joint; this has to be opened, and will probably need attention for about two months.

Erysipelas is sometimes observed round the navel of a new-born child, and when this happens the doctor's attention must be called to the matter immediately. The disease may occur in connection with vaccination, but only when cleanliness has been neglected. It is never found if the child's arm was surgically clean before the operation, and duly protected afterwards. If the mother cannot afford to buy a shield, a pad shaped like a hollow cone should be placed over the incision and held in position by a spica shoulder bandage.

Chronic erysipelas is usually found on the face and legs. The legs should be covered with cotton wool and bandaged.

Cold must be carefully avoided, and great care is necessary in washing an erysipelas patient.

The patient's room should, if possible, be kept in strict nursing order, as for all infectious fevers, and separate china, teacloth, bowl, etc., set apart for exclusive use of the sufferer.

### Incidents in a Nurse's Life.

Contributions to this column are invited.

### AN UNCOMMON EMERGENCY.

Now that we hear so much of maternity work, and the nursing papers are full of the most necessary and important precautions which both monthly nurses and midwives should use, with regard to the asepsis of the hands and all articles used during labour, and, in fact, during the whole period of the puerperium, I should like to give a short account of a rather uncommon emergency that occurred to me whilst nursing a midwifery case.

I remember during my probation at hospital having the chief, and perhaps most important, complication of child-birth—namely, hæmorrhage—suspended over my head like a veritable sword of Damocles. The care one should take to prevent an occurrence of the sort, and, if such a misfortune overtook one's patient, the best means for the immediate stopping of the flow of blood. I was very young at the time, and quite a beginner in the school of nursing. I vas nursing a lady who, not being in very affluent circumstances, had only engaged me for a fortnight. The labour for a primipara had been a quick and comparatively easy one; the placenta and membranes had come away in a perfect condition. The patient apparently made a speedy recovery, and the doctor allowed her to get up on the eleventh day; and from then until the day I was leaving I got her up for a short time in the afternoon. As there was still a slight discharge, I was particularly careful in not allowing my patient to walk about the room, which she was, after the first day or two, most anxious to do; the greatest exertion she took was to walk once round the bed on the thirteenth day. At four in the early morning of the day I was leaving I noticed, whilst attending to my patient, that there was more discharge than usual, and of a red colour. I kept her quiet, and there was no excessive flow, or indeed anything to be at all worried about, until I was going to my breakfast at eight o'clock. The discharge suddenly increased, and, although I did not think it was at all necessary to send for the doctor, I decided to give her a hot douche. This, however, did not stop the hæmorrhage, which now became rather alarming. On seeing this I immediately sent for the doctor. The uterus, curiously enough, was fairly well contracted. Having everything to my hand, I was enabled to give my patient another still hotter douche; I then, with the help of her mother, placed a pillow underneath her hips, thus raising her pelvis. I also wrung out a towel in cold water and placed it on the abdomen. The room being small, I was able to keep up a gentle friction on the uterus with one hand whilst I stretched with the other for things within my reach.

The patient's relatives were hopelessly helpless, and I could get no assistance; I had even a difficulty in getting anything hot for the patient's feet, as she was by this time in a state of considerable collapse. There was no hot-water bottle in the house, and we had to wrap one of the oven shelves in my flannel apron to do duty instead!

By this time the doctor luckily had arrived. He at once administered a dose of ergot. On making a digital examination he found the vagina full of clots. I forgot to mention that before his arrival two very large clots had been passed, after which the patient felt slight relief.

At this stage the patient being almost unconscious, we gave an ounce of brandy. Shortly after she was violently sick, and the faintness passed off.

The hæmorrhage having nearly ceased, the doctor left, promising to return in a few hours' time. Shortly after his departure a great quantity of clots were passed, and then hæmorrhage entirely ceased.

My patient was kept flat on her back, and allowed no solid food for two or three days. She was put on ergot and brandy three-hourly. Her recovery was satisfactory and rapid, and at the end of three weeks I was able to leave her quite safely. Of course, violent hæmorrhage, occurring as in this case at the end of the second week after confinement is very rare, and neither the doctor nor myself could discover the cause of it.

I have often thought how much better it would be if practitioners would entrust to their nurses' care a small quantity of ergot to be administered in cases of emergency.

Of course, looking back as I do now, I can see how much wiser I should have been to send for the doctor earlier, and not have undertaken so much responsibility.

But, like a good many young nurses, I was afraid of calling "wolf," and thus bringing upon myself the doctor's censure.

I am wiser now.

### In the Accident Ward.

The long-drawn, metallic clangour of the accident-gong sounds on the air. An awed, agitated murmur from a group of idlers at the gate of the hospital,—the regular tramp, tramp through the entrance and up the stone corridor of those who, with strained, anxious faces, are bearing their wounded workmate into the ever-open arms of the Palace of Pain.

Arrived at the top of the corridor, the leading man casts a hurried glance to right and left; but he needs not to hesitate, for a blue-coated porter stands there, and, with a quick glance at the covered stretcher, precedes them as guide to the accident-room.

A lofty, white-walled room, lighted by three windows and a domed skylight, its atmosphere faintly redolent of chloroform; four long, narrow tables, each covered with a thick blanket; several smaller ones, on which lie white, enamelled basins and trays; a huge, glass-walled cupboard, revealing the glitter of polished instruments within; a shirt-sleeved doctor bending over a washstand, vigorously removing from his hands traces of a recent "accident"; a trimly-uniformed nurse rearranging a dressing-table with deft, capable-looking fingers; a couple of porters apprised of the present occasion by the far-reaching tones of the accident-gong. Everywhere indications of preparedness, vigilance, efficiency.

The grim procession halts. Slowly, carefully, with the aid of the porters the occupant of the stretcher is transferred thence to one of the long tables. The doctor comes forward, wet-armed, alert-eyed, and asks a few concise questions. The leader pants out breathless, agitated replies—the transit of the burden has been in a measure laborious; moreover, the injured man was his "mate" in the work in which they were employed. His companions look down silently upon their stricken companion, and mop

### IN THE ACCIDENT WARD—*continued.*

heir foreheads with their grimy caps. The doctor signs dismissal, and, with a farewell, backward glance, they file quietly out, preceded as before by their guide.

The patient is quite conscious, a young, fair-haired man. Beneath the grime thrown there by his work his face shows white with the sickly pallor of pain, and his lips are tightly shut in a strong effort to keep back a groan. He looks up at the doctor with an expression of startled inquiry.

"'Tain't much, is it, sir?" he says anxiously, in a hoarse, weak voice.

"Now, don't you talk for a few minutes, my man," is the answer in the quiet, grave tones, which tell the porters that the case in hand is of a serious nature.

Then follows the examination; rapid, searching, and as painless as experience and skill can make it. At its conclusion the doctor turns to the sister, orders the admission of the case, and gives directions as to immediate treatment.

"Is I to stop in, sir?" comes faintly from the patient; and the doctor sees the blanched, suffering face looking earnestly at him.

"Yes, you must stop in; you know, you've had a nasty fall," the doctor answers; and he lays his hand gently, almost caressingly, on the forehead where great beads of sweat are gathering.

"It's all right for myself. I'm best kept in, I know; it's the missus I'm thinking of," continues the weak voice jerkily. "She'll be awful put about. But p'r'aps it won't be for long—eh, sir?"

The doctor looks down at the fair boyish face, and his lips tighten.

"No, it won't be for long," he says very gently.

The sister's eyes are dim.

. . . . . . . .

A small, neatly-dressed woman, bearing a baby in her arms, is standing hesitatingly at the crossing of two of the hospital corridors. Beneath her black sailor-hat the fragile, girlish face gleams strangely white, and her blue eyes have the startled, appealing look of a little child smarting from a blow received for an inscrutable reason.

A nurse emerges from a large door whose upper panels are composed of frosted glass, and, noting the forlorn, waiting figure, comes forward quickly.

"I've lost my way, sister," the woman says, in a low, toneless voice. "They told me, but I haven't rightly took it in. Where is he?"

"Whom are you looking for?" asks the nurse gently.

The dull anguish of the white face appeals to her.

"My husband, sister—Jack Haswell. They say he was brought here from the shipyard an hour ago."

"Oh yes; he is waiting for you," replies the nurse, and there is a note of relief in her voice. "But come in here first for a minute, will you?"

She precedes the woman into a small, barely-furnished waiting-room, and motions her to a chair. Mrs. Haswell sits down, slowly, wearily, but her eyes look out in the direction of the glass-panelled door, beyond which she has rightly divined her Jack lies.

There is silence for a minute; then—

"Mrs. Haswell," says the nurse gently—her young face is full of sympathy as she looks at that sorrow-stricken figure—"I am afraid——" She hesitates.

Mrs. Haswell turns quickly towards her.

"What is it, sister? I'd rather know. Is he badly hurt?"

The blue eyes set in the white, stunned face, are instinct with fiercely pathetic inquiry.

The nurse draws nearer, and puts her hand on the woman's shoulder.

"He is very, very ill, Mrs. Haswell," she answers.

That is all; but the wife has understood. She had caught in her breath whilst awaiting the reply; but it escapes now in a little shuddering moan of anguish. Her lips pucker as though in physical pain, and her grasp of the sleeping baby tightens. Then—

"Take me to him now," she says simply. Her voice has the unnatural, monotonous ring of one speaking in sleep. "We'd best be quick. I'll be quiet. I won't fret him."

The nurse regards her for a minute keenly.

"Then will you come with me?" she says, and leads the way. The woman follows with footsteps that seem produced against their will.

The ward is unusually quiet. During the last hour there has been much subdued stir around that screened-off bed in the darkened corner, but the grim fight between death and medical science has been swiftly decided. The house surgeon, with a frown on his forehead, has left, and only a nurse remains—watching, waiting.

Jack Haswell lies with closed eyes. His face is very white—almost as white as the pillow on which it rests—and the breath escapes from the blue-lipped, half-opened mouth in shallow, painful gasps.

Mrs. Haswell stands for a minute motionless at the foot of the bed, her gaze fixed—unseeingly, a bystander might think—on the piteous wreck of the stalwart young husband who had waved her a cheery good-bye four short hours ago. A little moan breaks from her, and with one hand she makes a vague, fumbling gesture in the air.

The nurse quickly places a chair at the bedside, and leads her to it.

The man's eyes open slowly—honest grey eyes, their pupils dilated with suffering. Into them springs a light of joyous recognition that triumphs momentarily over the pain.

"Why, it's Nellie," he says softly, dreamily. "I knew you'd come."

The woman cannot trust herself to speak. For his sake she must keep calm; but she puts one thin, toil-hardened hand beneath the bedclothes, and slips it into his which lies there so cold, so still.

The man speaks again—jerkily, painfully—

"Have they told you, Nell?"

The blue eyes and the grey meet, and in silence all is told, all understood.

"You must go back to your mother, Nell," the pained, tender voice goes on. "She'll be good—to you—an' the Grenadier." A faint smile flickers across his face as he utters the nickname given by them six months ago to the baby of whom both were so proud. "I'd like—to—kiss—our little lad."

The woman lays the sleeping baby face for a minute against his father's.

"Jack," she whispers brokenly, "my bonny Jack!"

That is all, but through the simple words throbs the fierce agony of a love powerless to hold.

"I can't talk—any more." (The woman has to bend low to catch the words, so faint are they.) "Good-bye, Nell; I'm glad—you got me to—go to chapel—with you. It's—all right."

. . . . . . . .

Half an hour later a heart-broken woman is led from the ward. A nurse follows, carrying "the Grenadier" in her arms. He coos gaily, and with feeble, fluttering fingers pulls at her muslin cap-strings.

### Visit to a Model Hospital in Switzerland.

BY AN ENGLISH MATRON.

It has seldom—I might even say never—fallen to my lot to visit a hospital which appears to me to have been planned with so much thought and care for the welfare of the children and for the convenience of the nurses as the Children's Hospital in Zurich, Switzerland, which I recently inspected. My experience, too, has not been a limited one, including as it does America, Canada, South Africa, Egypt, etc. I learnt that before this hospital had been built, some two or three years back, one of the Zurich professors had paid visits to many of the best children's hospitals in Europe, and that this institution was the result of his inspection, it having been built under his immediate supervision. The nursing question does not appear to be nearly so difficult in Switzerland as in France and Italy; many ladies of good family are going into hospitals for six months' training as "volunteers," paying a little for their maintenance, so that I quite believe the nursing profession will soon be as popular there as it is in England, and certainly the authorities appear to be encouraging it by providing more suitable accommodation for the nurses.

### The Sisters.

There was so much to see and so many points to ask questions about that I had not time to go fully into the nursing question even of that one hospital, but the tending of the sick was undertaken by sweet-faced and intelligent sisters of some religious order, not nuns, who, I was told, received no pay, but were fully trained in other hospitals before being appointed to the care of the children. They are comfortably housed, each having a separate bedroom, are fed and clothed, and provided for in a home in their old age. They are assisted by a few of the "volunteers" whom I have already mentioned. I think there are 130 cots in the hospital, including those in several small isolation blocks in the grounds for the various infectious diseases which might crop up in the wards.

### The Bath Arrangements.

Upon entering the hospital the porter's room and the secretary's office are on the left, and on the right an airy waiting-room, beyond which no new patients are allowed to go until they have been examined by one of the medical officers. If they are passed by him as suitable for admission and free from infectious disease, they next proceed to the bath-room close by; here, as all over the hospital, the baths and all utensils are so spotlessly clean, both the white enamel and all the "brights," that it is difficult to believe that all is not brand new, instead of having been in use already for a couple of years. There is a fixed big bath and a smaller one for babies; the latter is raised to a comfortable height for the nurse, and is on large rubber castors, so that when finished with it can be wheeled over a waste-pipe, and the water allowed to flow away from the hole in the bottom of the bath, to which a short pipe is attached. In the taps which supply all the baths are fixed thermometers, and it appears that you can regulate the heat of the water as it runs into the baths by the way you turn the handles of the taps. Into the walls of the bath-room a cupboard is let in, and here all the children's own clothes are kept in pigeon-holes, neatly labelled.

### Heating by Electricity.

We then proceeded to the wards. Here, as everywhere, the floors are of terrazzo, but down the middle of the ward a piece of washing oilcloth is let in, so that it is quiet and not too hard for the feet. The walls are white in a good washing paint with an enamelled surface, and there are suitable pictures for children painted as a frieze round the top. Most of the wards seemed to contain about 16 cots, but I believe that there were only eight in the babies' ward. In the wards themselves there are so many things to mention that it is difficult to know where to begin. The cots are of the most modern pattern and painted white; the bed-linen and the children's clothes are beautifully clean, and the children have a few toys to play with, and appear to be perfectly happy and contented. Hot and cold water are laid on everywhere, which always means much saving of labour. There are, however, no hot-water bottles to be filled; the babies, who require more warmth than that provided by a most carefully regulated and moistened ward temperature, are placed on a kind of double mackintosh or small water-bed, which is heated by electricity; the steam kettles and inhalers are also heated by electricity. I thought of the alarms we have when restless children have steam kettles heated by methylated spirit, and I wondered whether the linseed poultices are heated by the same means. Of course, all the corners of the wards are rounded, and the cupboards are all let into the walls, so that there are practically no ledges to collect dust, and the glass doors to the cupboards revealed the tidiness inside. I noted, too, the clean labels on the bottles, etc., which make such a difference to the smartness of a ward. In each ward there is a telephone for communication all over the hospital, and a lift to bring everything up from the kitchen; at the other end of the ward there is a lift-shoot to the basement, down which all dirty linen is immediately thrown and is sorted in the basement, so that nothing soiled is kept in or near the wards.

### Some Details of the Wards.

The wards are lofty, and I was puzzled to see a little staircase and a gallery with a doorway about half the height of the ward, in a corner near the dirty clothes shoot, but I found that it was a lavatory for the ward nurses, and beneath it on the same level as the ward is the lavatory for the children, with lilliputian fittings. There is a bath-room in each ward, and a small ward kitchen, with many little labour-saving contrivances worked by electricity, and the kitchen is so built that the nurse can see and hear all that is going on in the ward while she prepares the feeds or washes up. Above the ordinary electric lights there is a green-shaded lamp, which can be turned on for night use. There are large and sunny glass verandahs outside many of the wards, and most of the glass sashes are so made that they can be removed in the summer, and the children in their cots can practically live in the open air. Outside the glass sashes are sunblinds, so made that as you pull them down the cord automatically rolls itself up on a reel. The ward windows, or rather the double ventilators at the top, are opened by simply pulling down a lever which protrudes from the wall, so that there are no visible window cords to wrestle with and to collect the dust. As I went up the stairs I noticed little knobs on the rails at short intervals, to prevent the boys from attempting to slide down the banisters, and a little hand-rail placed at a convenient height for the small children to hold on to.

### The Theatre.

The notable feature of the babies' ward is the incubator, just then unoccupied, and near the heating apparatus there is a small cupboard in the wall, with a neat pile of sundries, all quite warm and ready for use, without having to hang them by the fire to warm—a particularly good arrangement, as there are no fires to hang them by! The theatre and $x$-ray rooms are as nice and complete as anyone could wish; everything necessary for aseptic surgery appears to be there, and to be kept in excellent order. I was also shown several large and bright private rooms, where children could be taken as paying patients; and, if the mother wishes, it can be arranged for her to stay in the hospital with the child.

The Gymnasium and Kitchen.

A visit to the basement only made one wish for time to explore more thoroughly. Here we glanced into a room nicely fitted up as a gymnasium, and were told that it was used, under medical direction, for children with tendencies to spinal curvature and other deformities. The kitchen is bright with much shining copper, and we observed that the lids of the huge cauldrons for soup and for potatoes, etc., are so arranged that when you begin to raise them they move up by themselves by means of an overhead chain and a pulley, and are safely held up until the inspection of the interior is finished. From this hasty visit I came away full of wonder at the avoidable inconveniences of many even of our newest English hospitals, though not without a hope that maybe other hospital authorities who are intending to build may see the wisdom of sending an intelligent observer to note the many good points of some of the more modern hospitals on the Continent. It was quite evident that the clever and kindly professor—to whose organising power the building of this hospital was chiefly due—had meant his countrymen to have the best that could be obtained, and the most severe critics would find it difficult to make with justice any really serious complaints.

### Rest of Mind.

BY A MATRON.

Rest of mind plays so important a part in the recovery of a sick person, from whatever kind of illness he may be suffering, that it is worth while for nurses to learn to observe signs of mental restlessness as distinct from mere physical irritability consequent upon the patient's actual bodily condition. Every nurse has had experience of cases that have had every care and attention lavished upon them, and yet from some mysterious reason they do not get on. It may be that some domestic trouble is worrying them, or they are uneasy about their own condition, or they have misunderstood the significance of some special treatment, or perhaps the tired brain is unable to sleep, and magnifies trifles which to a healthy person would be of no importance whatever. It is of little use telling a patient not to worry if there is real cause for anxiety. An observant nurse will usually be able, by a sympathetic manner and bearing, to gain her patient's confidence without any fussy attempts at asking questions. Sick people are like children; they know instinctively whom they can trust, and will often confide in the nurse if she shows real sympathy for their trouble. If unable immediately to relieve her patient's mind, she will be able to discover some method of conferring with his friends, or she will consult the doctor on his behalf, and, whatever be the outcome, the patient will be soothed and comforted by kindly human interest in his welfare.

Physicians and surgeons often determine to risk something in order to remove the dangerous anxiety which visibly hinders the improvement of the patient, but more often it is in the power of the nurse to calm the restless mind, and assure the patient that the doctor is quite satisfied with his progress, or she may be called upon to show grounds for hopefulness, and by her personal magnetism inspire her patient with courage in the face of an impending operation. What is more appalling to a nervous patient than the prospect of an operation? Here the optimistic nurse may be invaluable. She can dwell on the sunny side of a most serious surgical case, until the patient feels that it is not so very dreadful after all, and begins to look forward to a rapid recovery when the ordeal is over.

An interesting account recently appeared of a lady whose life depended upon an operation being performed within a given time, but whose condition of mental and moral collapse was such that the surgeons dared not undertake the case. At this juncture a friend, of a strong, hopeful character, visited the lady, and so inspired her with his own courageous spirit that her mental condition soon became completely changed, and, to the great surprise of her doctors, she submitted with hopefulness to undergo the operation, which was a great success. Such is the power which may be exercised by a strong nature over a weak, timid one.

A nurse should never convey by word or look her own fears about his condition to her patient; he will be morbidly sensitive to every depressing influence around him, and if he sees that the nurse despairs of his recovery he will lose all hope for himself, and thus may be deprived of the last chance of recovery. "While there is life, there is hope," is true of many cases, and much depends on the hopefulness of the patient himself. Slight causes often cause irritability of mind, and these, of course, should be promptly remedied by the nurse. A creaking door, a rattling window, a waving blind, a rustling skirt, or jingling keys—any of these things will jar upon a sensitive patient and keep him on the *qui vive*, listening for a repetition of the sound when he ought to be asleep. A really capable nurse will anticipate her patient's wishes and save him from every disturbing influence. Even folks who are well are often excited about trifling matters; how much more likely is the sick person, whose sense of proportion is not well-balanced, to become irritable if his food is brought to him half an hour late, or if the nurse has forgotten to execute some small commission which he has been feverishly waiting for. It is in the performance of little duties with as much thoroughness as great ones that the really good nurse is seen and known, and nothing that affects the well-being of her patient will be unimportant in her eyes. In the matter of sleeplessness the nurse may do much. Sometimes restlessness is a symptom of bed-clothes more than feverishness, and the removal of a heavy bed-quilt will give relief, or a cup of hot milk given late at night or in the early hours of morning will, by diverting blood from the brain to the stomach, soothe and often bring sleep to the patient. The tactful nurse, with a smooth, cool hand, may massage the forehead and scalp of a restless invalid with extraordinary effect, or if she has a quiet, even voice she may succeed in reading him to sleep. Sometimes the simple expedient of turning the pillow or changing his position will induce sleep. But, above all things, a nurse should cultivate a "restful, bedside manner," and this attractive qualification may be acquired by an observant woman as well as by the most skilful physician. It will be a valuable asset wherever her lot may be cast, and make her equally welcome in a royal palace or in the lowliest cottage. Hers it is to "soothe and sympathise" as well as to make beds and poultices.

### Danger from Burns and Scalds.

### EXAMINATION QUESTIONS FOR NURSES.

The question was as follows: (1) Define the different degrees of danger to life from various kinds of severe burns and scalds. (2) In a long and obstinately slow-healing injury from burns state what remedies you would apply and what measures you should take if allowed by the doctor to exercise your own judgment. N.B.—Observe carefully the difference between this question and that of last month.

### First Prize.

In cases of severe burns or scalds death may occur from shock, especially when the injuries are spread over a large surface, and particularly when the chest is involved.

Pyæmia caused by the absorption of pus may result in death. In the later stages death may take place from exhaustion.

In the case of a slow healing burn with much discharge I should apply boracic fomentations four hourly during the day, putting some other dressing on for the night. If the injured part were an arm or leg I should put it into a boracic bath 20 to 30 minutes twice a day until the discharge had stopped. Between the baths I should apply one of the following dressings:—

If the wound required stimulating lint wrung out of red lotion cut to the size of the wound, so as not to cover any new skin at the edges of the wound. Vaseline, zinc, or boracic ointments, sometimes any of these will do for a while, then a change is required. In such cases it is well not to keep on too long with one kind of dressing. For excessive granulations I should touch with caustic.

If none of these were successful I should ask the doctor's permission to use picric acid, a 5 per cent. solution, and pieces of lint wrung out and applied to the wound.

For a large wound it is best to use small pieces of dressing, they are less painful to remove.

Great care should be taken to prevent contraction of the skin during healing. "Dot."

### The Prize Winner.

"Dot" wins a prize because her paper is practical and to the point and does not go too much into details that belong more to the doctor's province than to that of the nurse. None of the remaining papers merits a prize; the question seems to have presented unexpected difficulties to the candidates.

### Honourable Mention.

This is only gained by three, as the standard reached by the papers is not so high as usual. The successful ones are "Few," "Ramleh," and "Central."

### Question for April.

What measures should you take to alleviate discomfort and avoid evil consequences in the case of a bedridden patient, male or female, who suffers from incontinence of urine. N.B.—You are to consider the house to be a poor one and appliances and comforts few.

The Examiner.

### Rules.

The competition is open to all. Answers must not exceed 500 words, and must be written on one side of the paper only, without divisions, head lines, or marginal notes. The pseudonym, as well as the proper name and address, must be written on the *same* paper, and *not on a separate sheet.* Papers may be sent in for 15 days only from the day of the publication of the question. All illustrations strictly prohibited. Failure to comply with these rules will disqualify the candidate for competition. Prizes will be awarded for the best two answers. Papers to be sent to "The Editor," with "Examination" written on the left-hand corner of the envelope.

In addition to two prizes honourable mention cards will be awarded to those who have sent in exceptionally good papers.

N.B.—The decision of the Examiner is final, and no correspondence on the subject can be entertained.

Any competitor having gained three prizes within the current year shall be disqualified from taking another until 12 months shall have expired since the first prize was gained.

### Everybody's Opinion.

[Correspondence on all subjects is invited, but we cannot in any way be responsible for the opinions expressed by our correspondents. No communication can be entertained if the name and address of the correspondent are not given as a guarantee of good faith, but not necessarily for publication. All correspondents should write on one side of the paper only.]

### THE TEACHING OF MASSAGE.

"A Teacher" writes: Will you allow me space for a few remarks on the teaching of massage? It seems a great pity that this valuable aid in connection with medical work is not better taught. What is the use of certificates when the students have not the proper movements and have not the slightest idea of the uses of these various movements. A student after going through a course with me stated that she had already been through a course of thirteen weeks' training at a well-known hospital. You may imagine my surprise when she had not the slightest idea of any movement save one—namely, "hucking" or "chopsticks." I feel most sorry for these students, whose time and hard-earned savings are thus wasted. This is not the first student of a like character I have had. My own experience was almost as bad. I studied under one of the oldest teachers of massage, or rather under the assistant teacher, and when I received my certificate I was not at all competent to take a case During the training I constantly complained of the teaching, stating I required thorough knowledge and not a piece of paper stating a lie. I fear that until good training is given such a valuable treatment will never receive the attention it should from medical men.

### CONVICTION FOR THEFT.

Alice May Nicholls (Hythe) writes: I was horrified on reading this week's Hospital to find that a nurse bearing the same name as myself had been accused of theft at Bow Street, found guilty, and sentenced to two months' hard labour. In my nursing career (extending over ten years) I have come in contact with many nurses. I was at the Bristol Private Hospital, Berkeley Square, Clifton; West Cornwall Infirmary, Penzance; the Royal Hants County Hospital, Winchester; and the City of London Lying-in Hospital, and for the last three-and-a-half years I have been district nurse here, Hythe, Kent, so I am sure you will understand how unpleasant it was to see someone with the same name in connection with such disgrace. Would it be troubling you too much to mention in your next week's issue that the Alice Nicholls mentioned is not myself.

Mrs. Alice Nicholls, of 11 Alexandra Villas, Brighton, writes: On reading your issue of the 7th inst. I find you have a paragraph on "Conviction of Theft." Now as the person mentioned has exactly the same name as mine I fear it is calculated to do me a great deal of harm, especially as it is known to many of my friends that I have stayed at the Nurses' Hostel and am also a nurse. I have not been out of Brighton since last January. Still all the people I know and may meet do not know that, so I am writing to ask you if you will kindly insert this in order to prevent any misleading statements being made.

### THE REGISTRATION OF NURSES.

"A Recently Qualified Nurse" writes: It was with great interest I read the suggestion of "H. P." to have a public examination. Every other profession has its examinations. The medical profession had theirs long before compulsory registration came into force. Why wait for Parliament? Would not an M.R.C.N. and an F.R.C.N. after the name solve the difficulty of the untrained nurses? The examination should, in my opinion, be open to all nurses who have had three years' hospital experience, or what the Board would consider an equivalent, with a testimonial from their matron, so that those nurses who have not the "Hall mark" of a three years' certificate, which include some of the best, should be free to enter. Among other advantages of such an examination would be: first, that intending probationers by a glance at "The Hospital's" pass-list would see the comparative value of the training schools. Secondly, there would be a uniform standard of knowledge required, and the hospitals which took little trouble in training their probationers would soon find it difficult to procure them. Thirdly, the small hospitals, provided the training was sufficient would find it easier to procure them. Here at this hospital we have lectures on theoretical and practical dispensing beside the usual subjects, and opportunities for learning sick-cooking, massage, Swedish exercises, electrical treatment, and $x$-ray work; also every nurse assists at from three to seven operations a week, and has daily clinical instructions from the house surgeons. The rules for the patients are few as the wards are small; many nurses from large institutions have said how much more like private nursing it was, and I have learnt more nursing in ten days here than in five months at a large hospital, where I had previously been, and yet the probationer discovers when it is too late that her certificate may be of no value on account of the number of beds.

### THE ROYAL BRITISH NURSES' ASSOCIATION AND REGISTRATION.

Miss Christina Forrest, Lady Superintendent of the Nursing Institute and Home Hospital, Bournemouth, writes: An extraordinary document is being circulated among the members of the Royal British Nurses' Association asking for votes for a new scheme of representation on the Central Board of the proposed Nursing Council. This scheme is the result of a conference of representatives of the Royal British Nurses' Association and the State Registration Society summoned by Mr. Tennant to see if a *modus vivendi* could not be arrived at in order to introduce into the House of Lords a Bill to which both Societies would agree. The deputation from the Royal British Nurses' Association, however, was not representative. It consisted only of the two medical men who are such well-known partisan advocates of the scheme for depriving the nurses of a proper representation on their governing body, and the Secretary of the Association—surely an anomalous position for the paid servant of a Corporation in which the majority of the members are known to differ very widely in opinion from the two medical men. The document asks the members to vote either for or against a new and objectionable scheme. If they vote against it, apparently it will be taken for granted that they approve of the Bill as drafted by the Royal British Nurses' Association executive committee, which is certainly not the case. Nothing short of a proportion of two-thirds of the members of the Council being trained nurses will satisfy the just demands of the nurse members of the Association.

### FEVER TRAINING IN GENERAL HOSPITALS.

"Hopeful" writes: Opinions have been asked in your columns on the affiliation of small hospitals with recognised training schools, therefore I venture to give mine and my reasons. I think, all things considered, that such an affiliation would be an advantage, both to matrons and nurses. Take the matter from the matron's side first. Hospital A. contains 300 beds, Hospital B. contains 26 beds. Now, if the matron of B. took probationers for a year as a preparatory course, with the understanding that they would be forwarded to A. for their training at the end of their preparatory year, the difficulty of obtaining probationers for B. would be considerably less. This rule would be specially favourable to probationers under age for training, or those whose health was not too robust, as the first year is often the most trying, owing to the complete change from ordinary life. The matron of B. favours this project. Now for the matron of A. She may prefer to have probationers without any previous experience—as some of our best training schools do—and initiate them under her own methods. This is where the difficulty comes in. Matron of A. may think previous experience more of a hindrance than a help, but if both hospitals were carried on under the same methods the arrangement ought to be successful without friction on either side. Naturally, nurses who are qualified, and go to small hospitals as staff nurses, do not care to do the menial work, such as dusting and scrubbing, which they did in their probationer days. They consider that the time has come when they should devote all their attention to the patients. Again, probationers will not enter a small hospital if they have any chance of admission to a large institution, so that the matron of B. has a great difficulty to face. Fever hospitals affiliated to general might also be worthy of consideration, so as to enable nurses desirous of fever experience to gain a little knowledge of the treatment of fevers. Six months at the least should be given to fever work, at whatever period of their training their matron deems suitable. Three years' certificated nurses enter fever hospitals as charge nurses, even though they may not have seen a case of fever. No doubt they quickly adapt themselves to the ways and methods; but how much more confident they would feel if they had enjoyed six months' fever training! Many of our general hospitals nurse enteric, and the nurses gain a sound knowledge of this particular fever; but what of the other types? I have been in two hospitals where no case of fever, even if one of the staff contracted it, would be nursed. In the hospital where I trained a block was set apart for diphtheria and enteric, for which I have since been thankful, as I took up fever nursing for some time immediately I completed my training. In this case the benefit applies to nurses only; but in the former—*i.e.* the affiliation of small and large hospitals, both matrons and nurses may reap the advantages. It is to be hoped that matrons will seriously consider the matter.

### NURSES AND THEIR GRIEVANCES.

"Gip" writes: May I now ask for a little space to reply to "A Clergyman's Daughter," who evidently has misunderstood my letter. I do not for one moment "think that I am the only one who has worries and anxieties." Much the reverse, for I am perfectly contented with my lot and love my work. As to meals, I have no cause whatever to grumble, being allowed twenty minutes at midnight and twenty minutes in the early morning. Therefore you will plainly see that I was not writing from a selfish point of view, but for the benefit of others. Evidently "A Clergyman's Daughter" has been training in some small hospital where "method" and "regularity" are not practised with so great efficiency as where I was trained; or perhaps the latter is one of the "spoilt girls." I hope that we shall soon be able to read more edifying letters in the correspondence page, which you have so kindly set aside for us.

"Albany" writes: I have read with much interest the letters in your paper on "Nurses and their Grievances," and I quite agree with the sentiments expressed in the letter from "A Clergyman's Daughter." It only proves that many women enter the nursing profession with the idea of its being play work, and such women are certainly best out of it. They lower the nursing standard, and are most prejudicial to the other members, whether in hospital or in private work. I was trained at a provincial hospital—120 beds; my first night duty was after nine months' probation, and was in the children's ward—seventeen beds—where I was on duty for fourteen weeks with two nights "off" during that time. It was a busy time, but a very pleasant one. Our hours were from 9 P.M. to 8 A.M. After six months' day nursing I was again "on night" in a men's surgical ward, and part of the time in the accident ward. I have no patience with these grumbling probationers. When I went first to hospital, and that is only five years ago, we had to polish the ward floor, and when on night duty, all the scrubbing to do, besides, of course, our temperatures, "fomentations," etc. I look back on my training as one of the happiest periods of my life, and though now married, my thoughts often wander back to my hospital days where, if one's heart is in one's work, the life is a very happy one. If one is a little tired, is not an expression of gratitude from a restless patient to whom one has perhaps given a drink of hot milk, smoothed the drawsheet, or performed some other little attention, even if it be only "That is a treat, nurse," enough to make one feel one is of some little use in the world.

"Clevedon" writes: I was so glad to read the letter from "A Clergyman's Daughter" on "Nurses and their Grievances," and so thoroughly agree with her that these grumbling nurses had far better take up some other work, and not degrade a profession like ours with such petty discussions and discontents. It is a marvel to me how they, seeing, as they must, such wonderful heroism and contentment amongst the poor in their wards—patients often suffering from overwork and lacking even the necessities of life—can have any inclination to think so much of their own long hours, their lack of easy chairs and comforts, and their little finger-aches. I have now a nursing home of my own, but often look back on my three years' training with regret, and would gladly go through some of its hard work again, to be rewarded by a word or smile from those poor things whose life is so hard, and who are usually so cheerful and ready to respond to any attempt to please or relieve them. In my training days the nurses were often apt to talk too much "shop." Now grumbling seems to have taken its place, both with hospital and a good many private nurses too, some of whom seem to be dreadfully afraid of a little bit of work. So much grumbling seems to me to lower the whole tone of nursing, and it is a wonder that girls, hearing of all these imaginary, or, at any rate, very exaggerated grievances, can ever make up their minds to enter hospital nowadays.

### TRAINED NURSES *v.* UNTRAINED WOMEN.

"A Fever Nurse" writes: I have been interested in the correspondence on the subject of outdoor uniform for nurses. I quite agree with your correspondents that something is really required in order to distinguish nurses from those make-believe individuals who masquerade in nurses' uniform, and I favour the idea of the hospitals adopting a special badge. But I really must take exception to the proposed exclusion of nurses of infectious hospitals. I fail to see why the line should be drawn there. One correspondent suggests that the necessity for nurses in infectious hospitals does not exist because they have to change their dresses before they come out. Quite so; but there is the economical side of the question. The "pretty pranks" of the few women to whom "St. Thomas's Nurse" refers is quite beside the point and offers no sound argument for a ban to be placed upon the whole body. I opine that even "St. Thomas's Nurse" would not claim that all nurses in general hospitals are above reproach. There are, of course, good and bad in every profession; and further, as regards hard work, surely it is not seriously suggested that nurses in general hospitals have the monopoly of this? I should like your correspondent to increase her knowledge of nursing by taking a six months' course in a fever hospital, and she would then perhaps be ready to admit that the duties of her sisters in fever hospitals are none the less exacting and responsible than those in general hospitals.

### Death in our Ranks.

On April 4 Miss Edith E. Baker, for many years on the staff of Guy's Institution, and from 1900 to 1905 the home nurse, died at her sister's house at East Dulwich, after a long illness. She was buried at Hastings on Monday afternoon, and the affection that was felt for her by her fellow nurses was testified by many beautiful flowers which were laid on her coffin. At the same time a memorial service was held in Guy's Hospital Chapel, which was attended by many of her friends.

### Appointments.

[No charge is made for announcements under this head, and we are always glad to receive and publish appointments. The information, to insure accuracy, should be sent from the nurses themselves, and we cannot undertake to correct official announcements which may happen to be inaccurate. It is essential that in all cases the school of training should be given.]

Bridport Dispensary and Cottage Hospital.—Miss Sophia Morris has been appointed matron. She was trained at the North Infirmary, Cork.

City Small-pox Hospital, Bierley Hall, Bradford.—Miss Jeannie Brooke has been appointed nurse matron. She was trained at Sheffield Royal Infirmary, and has since been sister at Bradford Eye and Ear Hospital.

Croydon Union Infirmary.—Miss May Minnie Whessell has been appointed ward sister. She was trained at St. Marylebone Infirmary, and has since been staff nurse at Margate Cottage Hospital and charge nurse at Maidstone Union Infirmary.

Derbyshire County Nursing Association.—Miss Edith M. Bridges has been appointed superintendent in affiliation with Queen Victoria's Jubilee Institute for Nurses. She was appointed Queen's nurse on January 1, 1902, and has worked in Liverpool and at Quedgeley, Gloucestershire.

Hartley Witney Union.—Miss Eliza Burt has been appointed superintendent nurse. She was trained at Manchester Union Infirmary, Crumpsall, and has since been sister in the same institute and charge nurse at the Park Hospital, Hither Green.

Manchester Royal Eye Hospital.—Miss Violet James has been appointed sister. She was trained at the Infirmary, Bury, where she was afterwards night sister, sister of women's wards, and sister of male and children's wards. She has since been sister at Rochdale Infirmary.

Maternity Hospital, Aberdeen.—Miss A. M. Beedie has been appointed matron. She was trained at the Withington Infirmary, Manchester, where she has since been sister. She has also been sister at Queen Charlotte's Hospital, London, and matron at the Hospital for Accidents, Somerton, Somerset. She holds the certificate of the Central Midwives Board.

Spittlesea Infectious Hospital, Luton.—Miss Howbrigg has been appointed staff nurse. She was trained at Hull Sanatorium.

Willesden Infirmary.—Miss Annie Foster has been appointed charge nurse. She was trained at Southwark Infirmary.

### Novelties for Nurses.

(By Our Shopping Correspondent.)

### A DELIGHTFUL SOAP.

(The Erasmic Company, Warrington.)

Erasmic soap is almost too well known to need a recommendation, but there is one peculiarity about it which daily becomes more marked. Those who have once used it find it quite impossible to use any other! In most scented soaps the perfume is aggressive; in "Erasmic" it is both fresh and refreshing, whilst the soap itself is so pure that it makes the process of washing a distinct pleasure.

### TRAVEL NOTES AND QUERIES.

By our Travel Correspondent.

Twelve Days in N. Devon (Puggy).—This would be my plan. Go straight to Lynmouth, which is more picturesque than Lynton. Route—train to Barnstaple and coach to Lynmouth, or train to Minehead and coach on. Put up at the "Bath" Hotel or "The Rising Sun." I fear you will find it expensive, but I think you can arrange for a week or ten days at the terms you suggest. I should stay there at least nine days, leaving three for Clovelly. There is so much of beauty around Lynmouth that you cannot do better than make it your headquarters. If you get Pearson's, Ltd., 1s. guide to "Ilfracombe and District" you will have full particulars as to excursions. To reach Clovelly go *via* Bideford. At Clovelly go to the "Red Lion"; it is a little the cheaper of the two inns. Write beforehand for a room. No, you cannot include Dartmoor; that will be for another time. North Devon roads are a little tiring for cyclists, being so steep; you must therefore go shorter distances. Thanks for enclosure; the money goes, you know, to our convalescent fund for nurses.

A Fortnight's Holiday for £6 (Old Maid).—I fear this cannot be done, on account of the initial expense of the journey. In Brittany I could make you a nice little tour, but it would cost 5s. a day, and the return journey would be £2. It leaves you no margin at all. Can you spare another £2 or £3? If not, how would you like a nice place on the North Cornish coast, which would come within your means. Let me hear again at once; perhaps we can arrange something at only a little heavier cost. I am so glad that my last advice proved so satisfactory.

May in the Isle of Man (Storrs).—It seems to me that it is likely to be cold there in May. I have a reliable address in Douglas I could give you if you decide for the Isle of Man. Write to Henry Cook and Sons, Ludgate Circus, E.C., and ask them about the boats. He will give you dates and hours of sailing. If you do not mind the long journey, I think Devonshire or Cornwall would be better in May. Let me hear again what you think, and I will give you addresses.

A Fortnight on the Rhine (Ignoramus).—It is much pleasanter to go by yourselves. The Polytechnic tours are good, I believe, and much cheaper than Cook's, but I do not recommend either unless you are very helpless people. You ask my help without telling me any particulars. Write to me at once, and tell me exactly how much you can each afford to spend in the fortnight, and I can arrange your little tour accordingly. I should suggest making Heidelberg the limit of your travels.

A Fortnight Abroad Alone (Hope).—You could not do better than the Bay of St. Malo if you are alone, there are so many excursions to be made, and all are easy ones. First-class return to St. Malo £2 12s. 6d., second return £2 1s. Write to Mrs. Dyott, Villa Olga, St. Malo on a prepaid return letter card, and ask her if she can take you in at the date you mention, and ask her terms. Use my name. You would find all you want there, and Mrs. Dyott's daughters would be companions for you. You can visit Mont St. Michel from St. Malo in a day, but it is pleasanter to stay the night.

Rules in Regard to Correspondence for this Section.—All questioners must use a pseudonym for publication, but the communication must also bear the writer's own name and address as well, which will be regarded as confidential. All such communications to be addressed "Travel Correspondent, 28 Southampton Street, Strand." No charge will be made for inserting and answering questions in the inquiry column, and all will be answered in rotation as space permits. If an answer by letter is required, a stamped and addressed envelope must be enclosed, together with 2s. 6d., which fee will be devoted to the objects of "The Hospital" Convalescent Fund. Ten days must be allowed before an answer can be published.

### Notes and Queries.

### REGULATIONS.

**The Editor is always willing to answer in this column, without any fee, all reasonable questions, as soon as possible.**

**But the following rules must be carefully observed.**

**1. Every communication must be accompanied by the name and address of the writer.**

**2. The question must always bear upon nursing, directly or indirectly.**

**If an answer is required by letter a fee of half-a-crown must be enclosed with the note containing the inquiry.**

*Infirmary Training.*

(15) I should feel greatly obliged if you could tell me of a Union Infirmary in or near Liverpool where they would take me as a probationer; and how long would my name be required to remain on their books before I could expect to enter the infirmary? I am 21 years of age.—*H. H.*

You are not eligible to enter a Union Infirmary as a probationer till you are 23. You might, however, write for particulars to the Matron of the Liverpool Workhouse Infirmary, Brownlow Hill, Liverpool; also to the Matron of the Mill Road Infirmary, Liverpool, and of the Birkenhead Union Infirmary. The length of time you have to wait depends upon the number of applicants.

*Training.*

(16) Kindly tell me if Mount Vernon Hospital, Northwood, is a good training hospital?—*E. A.*

The patients treated at Mount Vernon are all suffering from some form of consumption, so that a nurse cannot receive an all-round training there. But, if satisfactory, they can pass on to a general hospital.

*Lung Trouble.*

(17) One of our Sisters is threatened with lung trouble. Would it be possible for her to render her services at some sanatorium in return for staying there? She is an excellent nurse.—*Deaconess Una.*

Very probably some such arrangement might be made. Consult the list of sanatoria in "Medical Homes," obtainable from the Scientific Press, Limited, price 7d., post free, and write to the matrons of any suitable institutions.

*Training.*

(18) Where can I get a year's good general training and certificate? I am 24 years old. Is there a demand for fever-trained nurses?—*C. L. E.*

You can only get a year's training—which is of little value in the nursing world—by becoming a paying probationer. Fever training should be taken in addition to general training to be of value for private nursing.

*Dispensing.*

(19) I am a nurse with an Irish qualification, and am anxious to become a qualified dispenser.—*A. L. C.*

Write to the Pharmaceutical Society, 17 Bloomsbury Square, W.C.

*Maternity Work.*

(20) I am anxious to take up maternity work, but am too poor to pay fees. Could you kindly tell me if there is any hospital that gives free training to ladies?—*Mrs. G. E. D.*

No hospital gives free maternity training to ladies, but possibly the Association for Promoting the Training of Midwives, Dacre House, Dean Farrar Street, Westminster, or the Rural Midwives Association, 47 Victoria Street, S.W., might help you.

*Snoring.*

(21) Can you give me a remedy for snoring?—*Alma.*

As a nurse you should know that snoring may proceed from many different causes, and therefore it is impossible to give a universal "remedy."

*Sanitary Inspector.*

(22) Will you please tell me where I can obtain full particulars about becoming a lady sanitary inspector. I am a trained nurse?—*F. E. S.*

At the Sanitary Institute, Margaret Street, W.

*Midwifery Certificate.*

(23) Do you know of any way in which I, a trained maternity and general nurse, can qualify for the certificate of the Central Midwives Board without going into a hospital?—*Provincial.*

You need not go into a hospital provided you have had maternity training and pass the examination of the Central Midwives Board. The offices of the Board, where all information can be obtained, are at 6 Suffolk Street, Pall Mall, S.W.

The Scientific Press, Limited, publishes a little work, "How to Become a Certified Midwife," which would help you.

(24) Is a poor-law infirmary with 340 beds, a resident medical officer, and about fifty maternity cases a year a recognised training school from which nurses can obtain the certificate of the Central Midwives Board; and what constitutes a training school, please?—*Hopeful.*

Write to the Central Midwives Board, 6 Suffolk Street, Pall Mall, S.W., and inquire. A hospital to be recognised as a training school for general nursing must have over 100 beds and a resident medical officer, and give a three years' training. A list of these institutions recognised by the Central Midwives Board is published in "How to Become a Certified Midwife," published by the Scientific Press.

*English Hospitals in Cairo.*

(25) How can I gain admission into an English hospital in Egypt?

Write to the Matron of the Kasr-el-Ainy Hospital, Cairo.

*Riviera.*

(26) Will you kindly give me the address of the private nursing home in Nice; also any others on the Riviera?—*B. T. H.*

The Riviera Nurses' Co-operation, Nice, Matron Miss Warcup. The Nice Nursing Institute, Villa Pilatte, Avenue Disambrois, Nice; and the English Nurses' and Medical Home, Sunny Bank, Via Borgo Pescia, San Remo.

*Stammering.*

(27) Can you tell me how a patient can be cured of stammering, or if there is no cure at all?—*D. B. N.*

There are several systems by which persons profess to cure this misfortune, and undoubtedly improvement is often the result of their efforts. But you should consult a nerve specialist.

*Gall-stones.*

(28) I shall be grateful if you will tell me if turpentine and æther are generally considered useful in dissolving gall-stones?—*Alison.*

The treatment of anything so serious as gall-stones must lie in the hands of the medical man. Consult a doctor, and do not try any remedies on your own account.

*Maternity Work in Homes.*

(29) Can you inform me of any nursing homes or institutions which employ extra nurses (non-resident) in maternity work, when their usual staff are engaged, the nurse so employed paying a percentage on cases thus obtained?—*Ella.*

We know of no home which works on the lines you indicate. If you have general in addition to your maternity training, you might be helped by joining the Nurses' Co-operation, 8 New Cavendish Street, W. But your wisest plan would be to advertise in a nursing paper, stating your needs and adding that you would not object to paying good percentage.

( ) I am a trained and certificated maternity nurse. Please tell me if there is a good institution in London or suburbs where they employ outside nurses for private maternity work, or a reliable home that I could offer my services to?—*H. R.*

See answer to "Ella."

*Cut-throat Case.*

(30) I would be obliged if you would answer one or two questions in your columns. (1) A "cut-throat case" is admitted to hospital early in the day—does the doctor order a special nurse, or does the superintendent nurse ask the medical officer if he wishes a special nurse for the case? (2) Is it the custom to leave it for the night sister to ask for one?—*Puzzled.*

1. Such matters are usually arranged between the sister in charge and the doctor. 2. It very frequently happens in such a case that a special nurse is not wanted during the day, but that later developments show the need.

*Maternity and Midwife Cases.*

(31) I was engaged to nurse a maternity case for a month, and was also engaged to two other cases as midwife only. I had been with the nursing case ten days when I was sent for to attend one of the midwifery cases already described. My first patient refused to allow me to attend the midwifery patient, and said that I must either give her up or leave. I did the latter. Can I claim for a fortnight's board-wages in addition to the fee?—*Anxious.*

No; you broke your engagement, and could not expect any patient to allow you under the circumstances to be attending professionally any other case.

**Handbooks for Nurses.**

| | Post Free. |
|---|---|
| "A Handbook for Nurses." (Dr. J. K. Watson) ... | 5s. 4d. |
| "Nurses' Pronouncing Dictionary of Medical Terms" | 2s. 0d. |
| "Art of Massage." (Creighton Hale.) ... ... ... | 6s. 0d. |
| "Surgical Bandaging and Dressings." (Johnson Smith.) ... | 2s. 0d. |
| "Hints on Tropical Fevers." (Sister Pollard.) ... | 1s. 8d. |

Of all booksellers or of The Scientific Press, Limited, 28 & 29 Southampton Street, Strand, London, W.C.

### For Reading to the Sick.

### THE HIDDEN LIFE.

Blest is the hope that holds to God,
In doubt and darkness still unshaken,
And sings along the heavenly road
Sweetest when most it seems forsaken.
Blest is the time that in the Eye
Of God its hopeful watch is keeping,
And grows into eternity,
Like noiseless trees when men are sleeping.

*F. W. Faber.*

As the roots of a tree are out of sight, yet from them it derives its firmness and stability, so upon the hidden life of the Christian, that life which is out of the sight of other men, his firmness and stability depend; and as it is through the hidden roots that the nourishment is drawn up to the stem and branches, and the leaf continues green, and the tree does not cease from bearing fruit, even so in the Christian's life, that life which is "hid in Christ with God," lie the sources of his strength and of his spiritual prosperity.—*Archbishop Trench.*

All pain, sickness, weariness, distress, languor, agony of mind or body, whether in ourselves or others, is to be treated reverently, seeing in it our Maker's Hand passing over us; fashioning, by suffering, the imperfect or decayed substance of our souls. Every sorrow is a billow on this world's troublesome sea, which we must pass over on the Cross, to bear us nearer to our home. Each trouble is meant to relax the world's hold over us, and our hold upon the world, each loss to make us seek our gain in heaven.—*E. B. P.*

But constant discipline in unnoticed ways, and the hidden spirit's silent unselfishness, becoming the hidden habit of the life, give to it its true saintly beauty, and this is the result of care and lowly love in little things. Perfection is attained most readily by this constancy of religious faithfulness in all minor details of life, in the lines of duty which fill up what remains to complete the likeness to our Lord, consecrating the daily efforts of self-forgetting love.—*T. T. C.*

Every step onward is hailed with delight by the cloud of witnesses who have already washed their robes and made them white in the Blood of the Lamb. "Out of much tribulation"—safely out, never again to be bathed in that terrible baptism, never again to drink of that bitter cup, but to sit down under His banner Whose Name is Love, and serve Him gladly and unweariedly for evermore.—*Mrs. Campbell.*

Send out Thy Light, the way is dark before me,
The path Thy Love has moulded out for me;
Send out Thy Light, that I may see Thy Footsteps
Calming the waters of life's restless sea.
Send out Thy Light, the clouds are dark above me,
Gathering in tempest from the angry sea;
Send out Thy Light, that I may see the storm-drops
Which fall from the dear Hand, once pierced for me.
Send out Thy Light, and lead me, Father, lead me,
Beyond this darkness, sorrow, and unrest;
Send out Thy Light, and guide me, worn and weary,
To the calm shelter of my Saviour's Breast.

*Anon.*

